# Marketable and Banned Pesticides in Agriculture: Categorization, Simulation, and Crystallography Review

**DOI:** 10.3390/ijms252211885

**Published:** 2024-11-05

**Authors:** Grigorios L. Kyriakopoulos, Ioannis Sebos

**Affiliations:** 1 School of Electrical and Computer Engineering, National Technical University of Athens, Zografou Campus, 15780 Athens, Greece; 2 School of Chemical Engineering, National Technical University of Athens, Zografou Campus, 15773 Athens, Greece; isebos@mail.ntua.gr

**Keywords:** categories of pesticides, simulated pesticides, marketable and banned pesticides, clusters, calculation matrices, adjustment factors, crystallography

## Abstract

Pesticides are playing a dominant role in modern cultivation practices to increase agricultural production but are also criticized for environmental depletion and soil and underground water degradation in field applications. An imperative need for greener pesticides has emerged in alignment with new innovations in agrarian and agricultural practices. This study provides a comprehensive review of marketable and banned pesticides that have been applied in past times or are still in use in agriculture. The collected literature production disclosed 35 distinct pesticides that were identified either isolated or in mixtures and residues. These pesticides are primarily applied in agricultural fields, but some of them were also criticized for human implications. Then, these 35 pesticides were grouped into four categories: insecticides (18), herbicides (9), fungicides (6), and acaricides (2). Furthermore, their molecular types, chemical structures, pKa or log Kow values were presented. Based on their chemical structure, the pesticides were also organized into two domains: “marketable simulated” and “banned simulated”, representing 43% and 57% of total pesticides, respectively. The simulations were generated by linking the elemental composition of each pesticide in the corresponding category; therefore, three “marketable simulated” (the acaricides were not marketable representative) and four “banned simulated” were demonstrated. In addition, the calculation of “adjustment factors” (−0.33 up to +0.50) and the “as calculated/marketable (or banned) simulated pesticides” ratios (0.946 up to 1.013) enabled the identification of four clusters of homogeneous characteristics: cluster 1: “Insecticides, Fungicides, marketable”, cluster 2: “Herbicides, marketable”, cluster 3: “Insecticides, Fungicides, banned”, and cluster 4: “Acaricides, Herbicides, banned”. Subsequently, the composition of the elements of C and H enabled the crystallography characterization of only the “marketable” pesticides, not those that are “banned”, with compounds that have been already registered in the “Crystallography Open Database”. Conclusively, implications, challenges, and future research recommendations have been proposed.

## 1. Introduction

Since the early 2000s, there has been an ongoing and thriving research focus on issues of agricultural and environmental interest [1,2]. Among these studies, there is an emerging number of research studies concentrated on the rational use of pesticides in agricultural fields, supporting the increase in crop yields in order to meet the increasing food needs of humans also following a healthy lifestyle of dairy nutrition meals. This global nutrition trend is balanced out by the severe health implications caused by the excessive use of pesticides that runoff from the natural environment to the urban-built-living environment through the complicated food chain from farming to dish. The theoretical coverage of the Introduction section has been organized in a chronological evolution of relevant studies in terms of the two decades of the 2000s and 2010s, being also included in the form of the following two subheadings, accordingly. It can be noted that this overview refers only to those cases that refer to pesticides for agricultural use and an increase in crop yields, some of these pesticides (not all of them) also having their human health implications on human fertilization capabilities studied. In addition, this chronological overview can be also referred to in alignment with the following subsections of this study, which provide a more detailed and systematic chronological, experimental, in-field, or case-study overview of the relevant published studies.

### 1.1. Decade of the 2000s

The concerns of pesticide application attracted an emerging scientific interest. In such a study, the effect of paternal occupational exposures on fertilizing ability was the subject of a survey conducted among 836 couples who sought in vitro fertilization treatment. It was shown that paternal pesticide exposure decreased significantly the fertilization rates of the couples exposed [3]. From an agricultural perspective, it can be denoted by the study of Osuji and Braithwaite [4]. In this study, the authors targeted the crop yields produced by peanut plants under different rates of pesticides applied. It was denoted that although pesticides and fertilizers had greatly enhanced food production, their combined biochemical effects were not known in detail [4]. This study also denoted that different peanut seed protein yields per pot were associated with different pesticidal properties applied [4]. These studies disclosed that significant yield differences are caused by effectiveness variation in productivity, quantities of supplied N, as well as the selection of herbicides, insecticides, and fungicides.

### 1.2. Decade of the 2010s

The concern of pesticide application to human health in this decade, the 2010s, has attracted a wider scientific interest compared to the previous one. From an agricultural perspective, among the effects of organic fertilizers, there is a study on cow and chicken manure and a pesticide investigation on the palatability and physicochemical properties of cooked rice examined in the paddy field of Okayama University [5]. It was demonstrated that cooked rice from grown plants offered overall a similar eating quality either by applying an organic fertilizer or a chemical fertilizer and pesticide-applied plot. The palatability in the applied organic basal fertilizer, chemical top-dressed, and pesticide-applied plot was inferior (hardly affecting) to that in the chemical fertilizer and pesticide-applied plot. In addition, acceptable high-eating quality rice production in organic culture was determined by the nitrogen absorption of organic fertilizer at the ripening stage [5].

In a similar crop-based study, the effect of pesticides and plant nutrition on wheat crops was studied by utilizing a small plot split-plot designed field trial at the experimental site of Pannonia University, Keszthely, Hungary [6]. Treatments applied at increasing rates for both fertilizers and pesticide application caused a significant impact on the grain yield of wheat. While increased pesticide applications are linked to an increased N rate, it was shown that plant nutrition applications were more sensitive and affectionate on yield efficiencies in comparison with those of plant protection treatments [6]. Subsequently, plant and soil interrelations should refer to plant protection and nutrition impacts on wheat cultivation yields [6].

Other research studies of the same decade were focused on the content of nitrate in groundwater. From the employed simulation, it was shown that higher losses of nutrients in the open field depended on an excessive application of fertilizers and water compared with the crop requirements. In this study, which was conducted at a coastal area located in Central Italy, greenhouse minor water losses were recorded because of the greater water use efficiency [7]. The simulation results showed substantial losses in the open field compared to lower losses reported in the greenhouse. In the Mediterranean zone, in this study of Central Italy, heavy autumn and spring rainfalls caused extensive leaching of nitrates that were further detected in high groundwater concentrations [7]. In a similar study, it was pointed out that the negative effect on the capacity of soils to recycle nutrients is due to the presence of pesticides, with the modification of pesticide degradation rates through fertilizers, together with their non-targeted activity among soil microbial communities [8].

In the 2010s, it is also noteworthy that environmental alarming sensitivity emerged regarding the composition and the fate of marginal foods composed of different parts of plant foods that are discarded by consumers, including the peel, stalk, and leaves, even though this type of waste could possess a nutritional value. In the relevant literature, the critical point is for researchers to analyze the phenolic compound, flavonoid, polyamine, nitrate, and pesticide contents of parts of vegetables that are cultivated in conventional and non-conventional methods and are usually discarded after human consumption. In this context, solutions for improving the consumption of these waste fractions and simultaneously reducing urban solid waste generation were proposed [9]. In this study, the highest detected quantities of nitrates and organochlorine pesticides occurred at conventional cultivations, mainly papaya peel and lemon balm leaves. In addition, the discarded parts of plant foods, mainly the stalks, leaves, and peels, were primary sources of antioxidant and phenolic compounds [9].

At the same time, another important study on human-concerned fertilization was focused on resuming the effect of pesticides and other pollutants on oocyte quality. At the time of research production, it was already known that some pesticides are operating as endocrine disruptors, with an impact on fertility depending on the type of product and the stage of ovarian development. These pesticides can also persist for a long time in a body that suffers from disorders during childhood or adulthood [10]. These authors investigated the pesticides’ effect in a group of young women supported in in vitro fertilization (IVF) and whose oocyte cohorts revealed a very high proportion of oocytes with a centrally located granular cytoplasm (CLCG). In these cases, higher risk rates of early miscarriage had been observed in the first trimester of pregnancy [10].

The scope of this study is firstly to identify what are the commonly applied pesticides that are applied in agriculture but also those that affect agricultural production and human fertilization; secondly, to organize those reported pesticides in terms of their types: insecticides, herbicides, fungicides, acaricides; thirdly, to organize these pesticides in terms of human, non-human, activity, jointly with their grouping per organic or chemical characterization, thus disclosing a better understanding of the identity and the functionality of them.

This study contains the following sections: Section 2 in which the retrieved documents are presented, followed by Section 3: Results. In Section 3, pesticides were identified and grouped into four categories, showing also the molecular types and their chemical structures, further mentioning their use in agriculture and the human implications by demonstrating their chemical formulas. In addition, in Section 3, the calculation matrices and the per-category emerging “marketable simulated” and “banned simulated” pesticides have been accompanied by the “optimum matching” to crystallography characterization. The crystallography characterization has been based on what is already registered in the “Crystallography Open Database”, focusing only on the currently “marketable” pesticides, not those that are “banned”. For this, the “adjustment factors” and the four clusters of homogeneous characteristics have been also developed. Subsequently, in Section 4, the discussion has been organized into three subsections: Categorization, Simulation, and Crystallography Valuation (Section 4.1), Evolutionary and Transitional Characteristics of Greener Pesticides (Section 4.2), and Overview of Applications of Green Pesticides (Section 4.3). Finally, the key aspects, implications, and future research orientations have been succinctly addressed in the concluding section (Section 5).

## 2. Methods and Analyses

**Figure 1:**
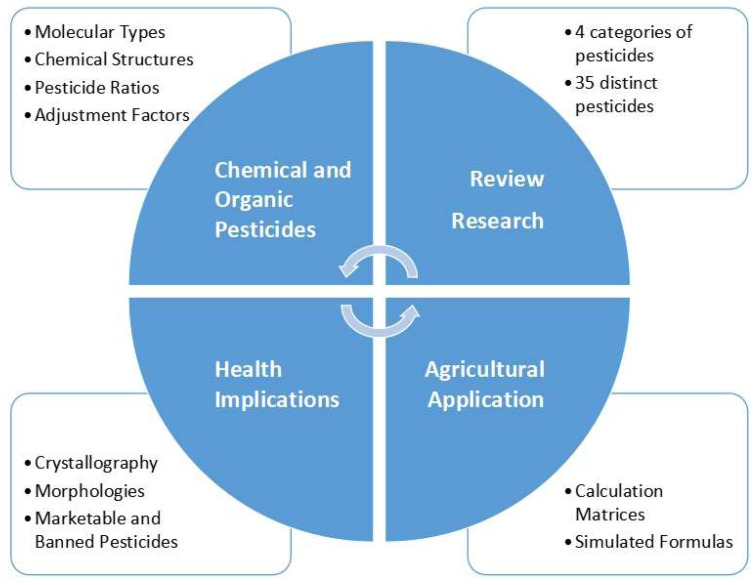
Research framework of this study. Source: authors’ own study.

The novelty of this study is the systematic investigation, categorization, and simulation of marketable and banned pesticides. Then, the crystallography characterization of only marketable pesticides (those that are in use for in-field agricultural applications) was further investigated considering their chemical and molecular characteristics. In addition, the reported pesticides were also valued not for the production maximization of their crops but also identified as potential determinants of human health hazards, which was also reported in the relevant literature. The research framework is shown in the following Figure 1.

Based on Figure 1, the source material consists of the documents retrieved from the relevant literature search at the Scopus database using relevant keywords. This research has focused on chemical and organic pesticides (upper part of Figure 1), with an interest in agricultural applications and health implications of the detected pesticides (ground part of Figure 1). The identification of 35 pesticides was accompanied by the organization of them into four categories, showing also the molecular types and the chemical structures of each identified pesticide. Then, a simulated formula of each one of the four categories of pesticides was developed, while these derived and “simulated” pesticides were further examined in alignment with the following:

- Their “marketable” or “banned” characterization in the relevant literature, as well as in the ongoing regulations and collective intergovernmental decisions inhibiting them for agricultural purposes. For the scope of this study, as well as due to the review type, the authors proceeded in the analysis of both marketable and the (up-to-date) banned pesticides in order to provide a complete profile of the “chemical”, not purely the “healthcare”, dimension of those studies that have been published during the last three decades of analysis. At this point, it is noteworthy that while the prohibition criteria are ongoing strictly to health implications, the joint presentation and evaluation of both marketable and banned pesticides enables readers to obtain a more comprehensive understanding of the compositional evolutionary characteristics per each one of these two domains since even the banned pesticides were used of in agriculture; thus, from a historic and chemical point of view, their co-presentation and co-evaluation with that of marketable, and potentially “greener” ones, make the analysis and findings more complete and comprehensive.
- Their formula matches with the already reported formulas in the Crystallography Open Database [11]. The “matching criteria” were (a) the equal number of elemental compositions of carbon (C) and hydrogen (H), (b) the presentation of, as much as possible, other elements in both the “simulated” categories of pesticides and the “Crystallography Open Database” [11].

The methods and analyses included in this study are the literature search outcome of documents (published in journals studies) retrieved from the Scopus database using the keywords “pesticides” and “fertilization” in their article titles. The “fertilization” term of this review study is considered only in the context of “human fertilization”, not in the context of “chemical fertilizers”. In addition, in the “collective” term of “pesticides”, each type of them is implied, such as insecticide, herbicide, fungicide, or acaricide. In Table 1, the key aspects of each retrieved study have been organized as “Pesticide/Pollutant” and “Aim–experimental–results”, accordingly.

## 3. Results

### 3.1. Identification and Clustering of Pesticides in Categories

Taking into consideration all the information contained in Table 1 above, it is noteworthy to mention the study of Naas and Saleh [18] in which it was stated the human implications of careless or hazardous consumption of fruits planted in greenhouses or in open fields in which cultivations contained pesticide residues, mainly in potatoes, fruits, and the eggplant [18]. This behavior can be attributed to the fact that farming common practices (at least in developing and economically emerging countries) have been related to the application of increased quantities of chemicals in fruits when harvesting, as well as to chemical pesticide application, showing long-term toxic residues in comparison with the crop-collecting periods. In this context, vegetarian consumers are recommended to consume fruits grown in local open fields and, simultaneously, to reduce hidden fruit consumption of those grown in greenhouses mainly due to the excessive use of pesticides and fertilizers for production/crop-increasing yields but are burdened from the high nitrates in fruits [18].

Another critical point of discussion is the reported differences between the use of fertilizers and pesticides during fruit growth, where the critical parameters were changes in sugar, amino acid, and organic acid composition [22]. Sugar concentration differences were enough for the consumers to be detectable in the study [22], where a sensory experiment was performed by preparing two orange juice samples in order to resemble the sweet/sour taste balance of juice from mandarin oranges, in which foliar fertilization was either applied or not. A non-trained individual test showed a percentage of 68% of respondents who correctly identified which juice had a sourer, or less sweet, taste. Subsequent studies could guide the impact of citrus growers, thus ultimately fostering the growth of the fruit with the desired superior sensory quality [22].

Summarizing the influential factors that led to the low level of the development of crop fertilization and pesticide application research should focus on promoting the development of crop fertilization and pesticide application machinery by deepening the integration of agricultural machinery and agronomy. The relevant research supported further the development of light and simple machinery and the promotion of mechanization towards the large-scale development of crop fertilization and pesticide application machinery, proving promising in the crops of sweet potatoes or similar ones [21].

In Table 2, the molecular type, the chemical structure, the IUPAC names, the corresponding categories, as well as the pKa or log Kow values of the 35 reported pesticides are presented. In addition, in Table 3, based on each retrieved document, the embodied information was further classified following the domains of human, non-human, organic, chemical, which were developed per each of the pesticides reported.

The mean value of the pKa values in Table 2 is 4.56, while the mean value of the log Kow values reported in Table 2 is 4.88. The calculations included the relevant pKa or log Kow values only once per pesticide; thus, in more than one value or in number ranges, then, only the average value was considered. From a functionality/physical meaning perspective, it is also noteworthy to mention that pKa is a method of determining an acid’s strength. A lower pKa value is correlated with a more powerful acid; thus, the lower pKa of an acid indicates that this acid dissociates more entirely in water. Regarding the pKa functionality in aqueous solutions, pKa is a measurable determinant of a molecule or ion capability to keep a proton, H^+^, at its ionization center(s). Subsequently, pKa refers to a chemical species’ capability towards ionization. This is also a metric for determining the strength of the hydrogen bonding as well as the attraction and the repulsive forces that are developed when explaining the sorption mechanisms, such as that of the adsorption of polluted adsorbates in carbonaceous and polymeric adsorbents [23,24,25,26]. The pKa values commonly refer to the literature, followed by specific temperatures reported.

**Table 3:** Profile information containing the domains: human, non-human, organic, chemical per pesticide reported. Source: synthesized by authors based on * [3,8,9,12,13,14,15,16,17,18,19,20,21].

Pesticide Type	Human	Non-Human	Organic	Chemical	Ref Item # in Table 1
Non-referred/specified	+	-	-	-	[3]
Non-referred/specified	+	-	-	-	[21]
Aldrin, atrazine, endosulfan, endrin, heptachlor, hexazinone, metribuzin, propachlor, simazine	-	+	+ Organochlorine herbicides	-	[12]
Endosulfan, DDD, DDE, DDT, dieldrin, endrin, HCB, HCH	-	+	+ Organochlorine pesticides	-	[14]
Aldicarb, aldrin, carbofuran, chlorpyrifos, DDT, diazinon, dieldrin, HCH, malathion	-	+	+ Organochlorine insecticides and organophosphate pesticides	+ Nitrates, as well as a group of characteristic chemical groups examined in the pesticide residue analysis of carbamates, organochlorines, organophosphates, and herbicides, including diazinon, malathion, carbofuran, aldicarb, aldrin, DDT, dieldrin, HCH, chlorpyrifos	[9]
Quinalphos	-	+	+ Organophosphate pesticide	-	[19]
Carbendazim, chlorpyrifos, Thiamethoxam	-	+	+ Organophosphate insecticide: chlorpyrifos	Benzimidazole fungicide: Carbendazim; neonicotinoid insecticide for chemical pesticides control: Thiamethoxam	[20]
Rice cultivation that was grown on organic fertilizer and pesticide-free plot	-	+	+	-	[13]
Malathion, methyl parathion	-	+	-	+	[16]
Difenoconazole, deltamethrin, ethofumesate	-	+	-	+	[8]
Bifenthrin, Carbendazim, Imidacloprid	-	+	-	+	[15]
Butachlor, Emamectin, Tebufenozide	-	+	-	Butachlor: acetanilide herbicide; Emamectin: avermectin family insecticide; Tebufenozide: molting hormone	[17]
Nitrates detection in greenhouse plant fruits using the mobile device of Green Test Eco.	-	+	-	+ Chemical pesticide-contained nitrates	[18]

* The explanation of symbols: +, the characteristic is referred to in the study, while, -, the characteristic is not referred/specified, or is out of scope, in the study. The studies in Table 3 are ordered by the following: firstly, human, then non-human; secondly organic (first entries in organochlorine pesticides, followed by that of organophosphate pesticides), then chemical; thirdly, the last six entries referred to mixtures of pesticides; thus, they have been ordered in terms of chronology of publication (the earlier published, first).

Based on Table 3 and the retrieved documents, the majority of pesticides are organochlorine herbicides and insecticides, followed by organophosphate insecticides. In addition, it is noteworthy that while the primary use of the reported pesticides is the increase in agricultural production and yields, not directly affecting humans, the ongoing monitoring of them suspected them as candidate substances that induce human implications and low fertilization rates in today’s societies worldwide. This is the reason that from the start period of the retrieved documents (being published from the early 2000s onwards) up to now, 43% of the referring pesticides remained marketable, while 57% of them have been banned, inhibiting them from in-field applications.

### 3.2. Calculation Matrices and Pesticide Simulations per Category

The procedure of generating the calculation matrices and deriving the simulation pesticides, which correspond to one per category: insecticides, herbicides, fungicides, and acaricides, is the following. A total of 35 retrieved pesticides have been grouped into the aforementioned four categories. Then, taking into consideration the molecular type of each category of the contained pesticides: 18 insecticides, 9 herbicides, 6 fungicides, 2 acaricides, the elemental composition of them has been collectively provided in Table 4, Table 5, Table 6, Table 7, Table 8, Table 9, Table 10, Table 11, Table 12, Table 13 and Table 14 per category. Subsequently, the general formula of the “simulated pesticide” per category has been typed as C_a_H_b_Cl_c_N_d_O_e_Br_f_F_g_P_k_S_t_, finding the indicators of a,b,c,d,e,f,g,k,t per pesticide category. In case fractional values have been derived, then, using the “adjustment factor”, a recalculation from fractional to integer numbers was received for the most frequently observed elements C and H.

Regarding the term “simulated pesticide”, there are two domains termed “marketable simulated pesticides” and “banned simulated pesticides” in order to better reflect the chemical analysis of this study that spans almost the last three decades, with the current and rapidly evolving trading situation of inhibiting the bargain of some of the past pesticides due to environmental and health implications restrictions/protection.

In addition, when the molecular type of each pesticide category C_a_H_b_Cl_c_N_d_O_e_Br_f_F_g_P_k_S_t_ was determined, then an attempt to associate as much of the simulated pesticide formula more precisely with the formulas that are compiled and listed in the open database of the “Crystallography Open Database” [11] was made.

Regarding the term “banned”, it covers the noncommercial launch in the relevant market based on the following two sources of banned pesticides: DG Health and Consumer Protection [27] and the Stockholm Convention on Persistent Organic Pollutants [28]. The Stockholm Convention on Persistent Organic Pollutants [28] is a global treaty between 186 countries aiming at protecting human health and the environment from chemicals that remain intact in the environment in the long run, thus widely distributed geographically, accumulating in the fatty tissue of humans and wildlife, and exerting harmful impacts on human health or the environment. The role of the Stockholm Conventions is to jointly tackle the life cycle of global chemicals and waste. According to the Stockholm Convention on Persistent Organic Pollutants (POPs) [28], the sometimes called “dirty dozen” of the 12 banned POPs are those of the following:

- Pesticides: aldrin, chlordane, DDT, dieldrin, endrin, heptachlor, mirex, and toxaphene;
- Industrial chemicals: hexachlorobenzene and PCBs;
- Unintentionally produced POPs: dioxins and furans.

In this study, it was perceived that for those pesticides that are not included in the two developed lists, which are “marketable” or “banned”, their permission or prohibition of using them in agriculture was reported by searching them on the web separately. Therefore, in cases where there is only partial use or inhibition in some specific countries (not in all), or in specific agricultural plantations (not in all), they are still assumed as tradable products, and then, they are characterized as “marketable”, not “banned”.

All calculation matrices are shown in Table 4, Table 5, Table 7, Table 8, Table 10, Table 11, and Table 13, while all matching formulas of the simulated pesticides with their crystallography information were retrieved from the depository of the Crystallography Open Database [11], and are shown in Table 6, Table 9, Table 12, and Table 14. The crystallography information covered only the marketable, not the banned, pesticides. The critical issue of these generated and “simulated” types per pesticide category is not to calculate all weighted indicators as absolute numbers, a, b, c, …, t, but to find the analogies, rations, and the identity of fractional composition in terms of the relative “weighted” presence of each indicator in alignment with the others. For this, the adjustment factor applied only to the first two elements C and H that correspond to the “hydrocarbon” part of the whole molecular structure.

**Table 4:** Calculation matrix of developing the “marketable simulated insecticide” from the 6 “marketable insecticides”. Source: authors’ own study.

Molecular Type of Marketable Insecticides (6 Counted)	C	H	Cl	N	O	Br	F	P	S
Elemental weighted indicators of the marketable simulated insecticide: C_a_H_b_Cl_c_N_d_O_e_Br_f_F_g_P_k_S_t_	a	b	c	d	e	f	g	k	t
Chlorpyrifos C_9_H_11_C_l3_NO_3_PS	9	11	3	1	3	0	0	1	1
Deltamethrin C_22_H_19_Br_2_NO_3_	22	19	0	1	3	2	0	0	0
Imidacloprid C_9_H_10_ClN_5_O_2_	9	10	1	5	2	0	0	0	0
Methyl parathion C_8_H_10_NO_5_PS	8	10	0	0	5	0	0	1	1
Quinalphos C_12_H_15_N_2_O_3_PS	12	15	0	2	3	0	0	1	1
Tebufenozide C_22_H_28_N_2_O_2_	22	28	0	2	2	0	0	0	0
Weighted indicators “as calculated”	13.67	15.5	0.67	1.83	3	0.33	0	0.5	0.5
Adjustment factor of rounding values (±) for C and H	+0.33	+0.50	-	-	-	-	-	-	-
**Molecular weights of**	**“As calculated” = 335** **“Marketable simulated” = 339.5** **“As calculated”/“Marketable simulated” ratio = 0.987**								

**Table 5:** Calculation matrix of developing the “banned simulated insecticide” from the 12 “banned insecticides”. Source: authors’ own study.

Molecular Type of Banned Insecticides (12 Counted)	C	H	Cl	N	O	Br	F	P	S
Elemental weighted indicators of the banned simulated insecticide: C_a_H_b_Cl_c_N_d_O_e_Br_f_F_g_P_k_S_t_	a	b	c	d	e	f	g	k	t
Aldicarb C_7_H_14_N_2_O_2_S	7	14	0	2	2	0	0	0	1
Aldrin C_12_H_8_C_l6_	12	8	6	0	0	0	0	0	0
Bifenthrin C_23_H_22_ClF_3_O_2_	23	22	1	0	2	0	3	0	0
DDD (p,p’) C_14_H_10_Cl_4_	14	10	4	0	0	0	0	0	0
DDT (p,p’) C_14_H_9_Cl_5_	14	9	5	0	0	0	0	0	0
Dieldrin C_12_H_8_Cl_6_O	12	8	6	0	1	0	0	0	0
HCH C_6_H_6_Cl_6_	6	6	6	0	0	0	0	0	0
Heptachlor C_10_H_5_Cl_7_	10	5	7	0	0	0	0	0	0
Mirex C_10_Cl_12_	10	0	12	0	0	0	0	0	0
Toxaphene C_10_H_8_Cl_8_	10	8	8	0	0	0	0	0	0
Malathion C_10_H_19_O_6_PS_2_	10	19	0	0	6	0	0	1	2
Thiamethoxam C_8_H_10_ClN_5_O_3_S	8	10	1	5	3	0	0	0	1
Weighted indicators “as calculated”	11.33	9.92	4.67	0.58	1.17	0	0.25	0.08	0.33
Adjustment factor of rounding values (±) for C and H	−0.33	+0.08	-	-	-	-	-	-	-
**Molecular weights of**	**“As calculated” = 356.4** **“Banned simulated” = 352.5** **“As calculated”/“Banned simulated” ratio = 1.011**								

Based on the aforementioned calculation matrix, the marketable simulated insecticide, counting for six marketable insecticides that have been reported in the literature, is C_14_H_16_Cl_0.67_N_1.83_O_3_Br_0.33_P_0.5_S_0.5_ (Table 4). Based on the aforementioned calculation matrix, the banned simulated insecticide, counting for 12 banned insecticides that have been reported in the literature, is C_11_H_10_Cl_4.67_N_0.58_O_1.17_F_0.25_P_0.08_S_0.33_ (Table 5).

In addition, it can be denoted that these calculations complied with the following preconditions:

- Due to the extreme (regarding the very high elemental composition of C, H, O compared to the other reported insecticides) case of the insecticide Emamectin (B1b) having molecular type: C_48_H_73_NO_13_, it was considered an outlier insecticide; thus, it was not counted in the molecular simulations.
- The adjustment factor was applied in order to convert the fractional weights to integer ones firmly and only for the first (for C) and for the second (for H) elemental weights, respectively.
- Searching for a compound that has been literature-reported, with the core part of the marketable simulated insecticide C_14_H_16_-, it was observed in the Crystallography Open Database [11].

It is noteworthy that while some of the 18 literature-reported insecticides contained the F element, in the marketable simulated insecticide (developed from the 6 marketable ones), there is not the F element in the derived marketable simulated insecticide, but it is contained in the banned simulated insecticide. It is also noteworthy that while some of the 18 literature-reported insecticides contained the Br element, in the banned simulated insecticide (developed from the 12 banned ones), there is not the Br element in the derived banned simulated insecticide, but it is contained in the marketable simulated insecticide.

Considering the formula of the marketable simulated insecticide, the compounds that contain the co-existing elements of C, H, Cl, N, O, Br, P, S in different fractions among various weighted indicators are provided below in Table 6. Therefore, seven out of eight total elements (C, H, Cl, N, O, Br, P) of the marketable simulated insecticide C_14_H_16_Cl_0.67_N_1.83_O_3_Br_0.33_P_0.5_S_0.5_ are also contained in the compounds (formulas) of Table 6.

**Table 6:** Crystallography information regarding the marketable simulated insecticide. Source: based on the Crystallography Open Database [11].

COD ID	Links	Formula	Space Group	Cell Parameters	Cell Volume
1501607	CIF	C_14_ H_16_ Cl N_5_	P 1 21/n 1	9.627; 4.9751; 35.2533 90; 95.79; 90	1679.9
1502021	CIF	C_14_ H_16_ N_2_ O_2_	P 21 21 21	8.466; 9.68; 16.183 90; 90; 90	1326.2
1502493	CIF	C_14_ H_16_ N_2_ O_3_	P 1 21/c 1	11.641; 13.621; 8.8 90; 99.28; 90	1377.1
1503839	CIF	C_14_ H_16_ Br N O_3_	P 21 21 21	5.5253; 8.5106; 30.7793 90; 90; 90	1447.35

**Table 7:** Calculation matrix of developing the “marketable simulated herbicide” from the 5 “marketable herbicides”. Source: authors’ own study.

Molecular Type of Marketable Herbicides (5 Counted)	C	H	Cl	N	O	P	S
Elemental weighted indicators of the marketable simulated herbicide: C_a_H_b_Cl_c_N_d_O_e_P_k_S_t_	a	b	c	d	e	k	t
C_17_H_26_ClNO_2_	17	26	1	1	2	0	0
C_13_H_18_O_5_S	13	18	0	0	5	0	1
C_3_H_8_NO_5_P	3	8	0	1	5	1	0
C_12_H_20_N_4_O_2_	12	20	0	4	2	0	0
C_11_H_14_ClNO	11	14	1	1	1	0	0
Weighted indicators “as calculated”	11.2	17.2	0.4	1.4	3	0.2	0.2
Adjustment factor of rounding values (±) for C and H	−0.2	−0.2	-	-	-	-	-
**Molecular weights of**	**“As calculated” = 246** **“Marketable simulated” = 243.4** **“As calculated”/“Marketable simulated” ratio = 1.011**						

**Table 8:** Calculation matrix of developing the “banned simulated herbicide” from the 4 “banned herbicides”. Source: authors’ own study.

Molecular Type of Banned Herbicides (4 Counted)	C	H	Cl	N	O	P	S
Elemental weighted indicators of the banned simulated herbicide: C_a_H_b_Cl_c_N_d_O_e_P_k_S_t_	a	b	c	d	e	k	t
Atrazine C_8_H_14_ClN_5_	8	14	1	5	0	0	0
Endrin C_12_H_8_Cl_6_O	12	8	6	0	1	0	0
Metribuzin C_8_H_14_N_4_OS	8	14	0	4	1	0	1
Simazine C_7_H_12_ClN_5_	7	12	1	5	0	0	0
Weighted indicators “as calculated”	8.75	12	2	3.5	0.5	0	0.25
Adjustment factor of rounding values (±) for C and H	+0.25	0	-	-	-	-	-
**Molecular weights of**	**“As calculated” = 253** **“Banned simulated” = 259** **“As calculated”/“Banned simulated” ratio = 0.977**						

Based on the aforementioned calculation matrix, the marketable simulated herbicide, counting for five marketable herbicides that have been reported in the literature, is C_11_H_17_Cl_0.4_N_1.4_O_3_P_0.2_S_0.2_ (Table 7). Based on the aforementioned calculation matrix, the banned simulated herbicide, counting for four banned insecticides that have been reported in the literature, is C_9_H_12_Cl_2_N_3.5_O_0.5_S_0.25_ (Table 8).

In addition, it can be denoted that these calculations complied with the following preconditions:

- There are no extreme values (regarding a very high or very low elemental composition among the reported herbicides); thus, no outliers were counted in this molecular simulation.
- The adjustment factor was applied in order to convert the fractional weights to integer ones firmly and only for the first (for C) and for the second (for H) elemental weights, respectively.
- Searching for a compound that has been literature-reported, with the core part of the marketable simulated herbicide C_11_H_17_-, it was observed in the Crystallography Open Database [11].

It is also noteworthy that while some of the nine literature-reported herbicides contained the P element, in the banned simulated insecticide (developed from the four banned ones), there is not the P element in the derived banned simulated herbicide, but it is contained in the marketable simulated herbicide.

Considering the formula of the marketable simulated herbicide, the following compounds, having also the remaining elements of C, H, Cl, N, O, P, S (C, H, Cl, N, O, S) in various weighted indicators, are provided below in Table 9. Therefore, six out of seven total elements of the marketable simulated herbicide C_11_H_17_Cl_0.4_N_1.4_O_3_P_0.2_S_0.2_ are also contained in the compounds (formulas) of Table 9.

**Table 9:** Crystallography information regarding the marketable simulated herbicide. Source: based on the Crystallography Open Database [11].

COD ID	Links	Formula	Space Group	Cell Parameters	Cell Volume
1503553	CIF	C_11_ H_17_ Cl O_2_	P 1 21/c 1	8.0876; 14.5181; 10.8895 90; 122.497; 90	1078.4
1504055	CIF	C_11_ H_17_ N O_3_	P -1	6.6599; 9.8956; 9.9738 108.604; 96.856; 97.248	609.01
1504903	CIF	C_11_ H_17_ N O_5_	P 1 21 1	6.9222; 8.7421; 10.5953 90; 103.477; 90	623.51
1506952	CIF	C_11_ H_17_ Br O_4_	P 32 2 1	7.208; 7.208; 42.0575 90; 90; 120	1892.36
1518628	CIF	C_11_ H_17_ Cl F N O Pt S	P 1 21/c 1	5.9315; 12.1005; 19.7 90; 103.067; 90	1377.34

**Table 10:** Calculation matrix of developing the “marketable simulated fungicide” from the 4 “marketable fungicides”. Source: authors’ own study.

Molecular Type of Marketable Fungicides (4 Counted)	C	H	Cl	N	O
Elemental weighted indicators of simulated fungicide: C_a_H_b_Cl_c_N_d_O_e_	a	b	c	d	e
Difenoconazole C_19_H_17_Cl_2_N_3_O_3_	19	17	2	3	3
Putrescine C_4_H_12_N_2_	4	12	0	2	0
Spermidine C_7_H_19_N_3_	7	19	0	3	0
Spermine C_10_H_26_N_4_	10	26	0	4	0
Weighted indicators “as calculated”	10	18.5	0.5	3	0.75
Adjustment factor of rounding values (±) for C and H	0	+0.5	-	-	-
**Molecular weights of**	**“As calculated” = 210.75** **“Marketable simulated” = 222.75** **“As calculated”/“Marketable simulated” ratio = 0.946**				

**Table 11:** Calculation matrix of developing the “banned simulated fungicide” from the 2 “banned fungicides”. Source: authors’ own study.

Molecular Type of Banned Fungicides (2 Counted)	C	H	Cl	N	O
Elemental weighted indicators of simulated fungicide: C_a_H_b_Cl_c_N_d_O_e_	a	b	c	d	e
Carbendazim C_9_H_9_N_3_O_2_	9	9	0	3	2
HCB C_6_Cl_6_	6	0	6	0	0
Weighted indicators “as calculated”	7.5	4.5	3	1.5	1
Adjustment factor of rounding values (±) for C and H	+0.5	+0.5	-	-	-
**Molecular weights of**	**“As calculated” = 294.33** **“Simulated” = 290.33** **“As calculated”/“Simulated” ratio = 1.013**				

Based on the aforementioned calculation matrix, the marketable simulated fungicide, counting for four marketable fungicides that have been reported in the literature, is C_10_H_19_Cl_0.5_N_3_O_0.75_ (Table 10). Based on the aforementioned calculation matrix, the banned simulated fungicide, counting for two banned fungicides that have been reported in the literature, is C_8_H_5_Cl_3_N_1.5_O (Table 11).

In addition, it can be denoted that these calculations complied with the following preconditions:

- There are no extreme values (regarding a very high or very low elemental composition among the reported fungicides); thus, no outliers were counted in this molecular simulation.
- The adjustment factor was applied in order to convert the fractional weights to integer ones firmly and only for the first (for C) and for the second (for H) elemental weights, respectively.
- Searching for a compound that has been literature reported, with the core part of the simulated fungicide C_10_H_19_-, it was observed in the Crystallography Open Database [11].

The following compounds, having also the co-existing elements of C, H, Cl, N, O in various weighted indicators, are provided below in Table 11. Therefore, all five out of five total elements (C, H, Cl, N, O) of marketable simulated fungicide C_10_H_19_Cl_0.5_N_3_O_0.75_ are also contained in the compounds (formulas) of Table 12.

**Table 12:** Crystallography information regarding the simulated fungicide. Source: based on the Crystallography Open Database [11].

COD ID	Links	Formula	Space Group	Cell Parameters	Cell Volume
1503874	CIF	C_10_ H_19_ Cl N_4_ O_2.333_	P 1 21/c 1	13.3425; 25.354; 13.6808 90; 116.203; 90	4152.4
1517717	CIF	C_10_ H_19_ Cl_2_ Cu N_3_ O_10_	P 1 21/c 1	9.1118; 9.2782; 22.1845 90; 100.041; 90	1846.78

**Table 13:** Calculation matrix of developing the “banned simulated acaricide” from the 2 “banned acaricides”. Source: authors’ own study.

Molecular Type of Banned Acaricides (2 Counted)	C	H	Cl	N	O	S
Elemental weighted indicators of simulated fungicide: C_a_H_b_Cl_c_N_d_O_e_S_t_	a	b	c	d	e	t
Carbofuran C_12_H_15_NO_3_	12	15	0	1	3	0
Endosulfan C_9_H_6_Cl_6_O_3_S	9	6	6	0	3	1
Weighted indicators “as calculated”	10.5	10.5	3	0.5	3	0.5
Adjustment factor of rounding values (±) for C and H	+0.5	+0.5	-	-	-	-
**Molecular weights of**	**“As calculated” = 314.00** **“Banned simulated” = 320.50** **“As calculated”/“Banned simulated” ratio = 0.980**					

**Table 14:** Calculation and marketable simulation results. Source: authors’ own study.

Pesticide Category	Number of Marketable Simulated Compounds	Adjustment Factor * for (C, H), Round Up to the Closest Integer (±)	Molecular Type of Simulated Compound	As Calculated/ Marketable Simulated Ratio (in Descending Order)
Herbicides	5	(−0.20,−0.20)	C_11_H_17_Cl_0.4_N_1.4_O_3_P_0.2_S_0.2_	1.011
Insecticides	6	(+0.33,+0.50)	C_14_H_16_Cl_0.67_N_1.83_O_3_Br_0.33_P_0.5_S_0.5_	0.987
Fungicides	4	(0, +0.50)	C_10_H_19_Cl_0.5_N_3_O_0.75_	0.946

*: The adjustment factor is a determinant of deviation and quantification regarding how dispersed the real molecular weight is from the simulated molecular weight.

Based on the aforementioned calculation matrix, the banned simulated acaricide (counting for two banned acaricides that have been reported in the literature) is C_11_H_11_Cl_3_N_0.5_O_3_S_0.5_. However, since the review study has been focused only on the marketable pesticides, not those that are banned, no further correlation between the core part of the “banned simulated acaricide” C_11_H_11_- with the Crystallography Open Database was made [11].

## 4. Discussion

### 4.1. Categorization, Simulation, and Crystallography Valuation

The main findings of the aforementioned calculation matrices and the accompanying crystallography correlations are the following: Firstly, for the majority of the simulated pesticides, the relevant multipliers in order to recalculate the fractional towards the corresponding integer weighted indicators were found. In all four categories: insecticides (18), herbicides (9), fungicides (6), and acaricides (2), there was a more targeted functioning among the emerging literature-reported types of pesticides, thus enabling a more successful matching and correlation to compounds registered in the Crystallography Open Database [11]. Moreover, regarding the derived simulated formulas with the formulas that are already open and accessible from the “Crystallography Open Database”, the majority of simulated/calculated formulas corresponded to realistic, already synthesized (or could be feasibly fabricated) compounds in order to be applied as marketable pesticides per each one of the three marketable categories: insecticides (6), herbicides (5), fungicides (4).

In addition, the aforementioned crystallography review, the calculation, and the simulation review of this study have been collectively presented in Table 14 and Table 15.

Based on Table 14, it was observed that the heaviest and the lightest formulas of the marketable simulated pesticides—those of insecticides and fungicides, respectively—were associated with positive adjustment factors of the elemental composition in C and H, ranging from 0 to +0.50. Contrarily, the moderate molecular weighted formulas were those of the marketable simulated herbicides, which sustained a negative adjustment factor of −0.20. It is also noteworthy that the total number of marketable pesticides, 15, is almost 43% of the total number of reported pesticides, 35. This observation can be attributed to the fact that there is a need for a long run time from the commercial launch of new types of pesticides to be tradable prior to the deleterious consequences to human health to be reported, as well as a plethora of different types of cultivations and plantations in which the same pesticides are used worldwide.

In an attempt to categorize the reported pesticides in terms of their “As calculated/Marketable simulated” ratios, two clusters can be identified: The pair of cluster 1: “Insecticides, Fungicides, marketable” is shaped due to the close but lower to 1 ratio value. Contrarily, cluster 2: “Herbicides, marketable” should be also categorized as separate and distinct, having a ratio value close to but more than 1. It is also noteworthy that the number of reported compounds for recognizing and categorizing cluster 1 is exactly two times higher than the number of reported compounds for cluster 2.

**Table 15:** Calculation and banned simulation results. Source: authors’ own study.

Pesticide Category	Number of Banned Simulated Compounds	Adjustment Factor * for (C, H), Round Up to the Closest Integer (±)	Molecular Type of Simulated Compound	As Calculated/ Banned Simulated Ratio (in Descending Order)
Fungicides	2	(0, +0.5)	C_8_H_5_Cl_3_N_1.5_O	1.013
Insecticides	12	(−0.33, +0.08)	C_11_H_10_Cl_4.67_N_0.58_O_1.17_F_0.25_P_0.08_S_0.33_	1.011
Acaricides	2	(+0.5, +0.5)	C_11_H_11_Cl_3_N_0.5_O_3_S_0.5_	0.980
Herbicides	4	(+0.25, 0)	C_9_H_12_Cl_2_N_3.5_O_0.5_S_0.25_	0.977

*: The adjustment factor is a determinant of deviation and quantification regarding how dispersed the real molecular weight is from the simulated molecular weight.

Based on Table 15, the opposite situation between the marketable and the banned pesticides was observed. In particular, the heaviest and the lightest formulas of the banned simulated insecticides and fungicides, respectively, were associated with positive adjustment factors of the elemental composition in C and H. Contrarily, the moderate-to-heavy molecular weighted and banned simulated acaricides and herbicides were associated with overly positive adjustment factors, ranging from 0 to +0.50 but also with lower, close to but lower than 1 “As calculated/Banned simulated” ratios. In this “banned simulated pesticides” domain, there should also identified two clusters: cluster 3: “Insecticides, Fungicides, banned”, cluster 4: “Acaricides, Herbicides, banned”. However, it is noteworthy that the total number of banned pesticides, 20, is almost 57% of the total number of reported pesticides, 35. This observation can be attributed to the fact that the reported documents of pesticide retrieval were internationally covered and also expanded to a widely dispersed and diversified spectrum of cultivations and plantations, thus necessitating a decades-level period prior to the deleterious consequences to human health being reported.

In such a way, rational use of these synthesized “greener” pesticides towards precise agriculture and widely sustainable development could be realistic, in alignment with the better preservation of natural sources and precautionary/preventive, rather than post-treatment, human health.

### 4.2. Evolutionary and Transitional Characteristics of Greener Pesticides

Based on the analytical and methodological structure and findings of this study, the highly liquefied and transitional characteristics of the pesticide marketplace can be argued. Persistent organic pollutants (POPs) are a class of highly hazardous chemical pollutants that are a serious, global threat to human health and ecosystems. POPs are substances that specifically (a) remain intact for exceptionally long periods of time (many years), (b) become widely distributed throughout the environment as a result of natural processes involving soil, water, and, most notably, air, (c) accumulate in living organisms including humans and are found at higher concentrations at higher levels in the food chain, and (d) are toxic to both humans and wildlife [28]. Some POPs are pesticides, some are industrial chemicals, and some are unintentionally produced by-products that form during certain combustion and chemical processes. Many of these chemicals benefit pest and disease control, crop production, and industry [28].

Unintentionally produced chemicals include dioxins, which result from some industrial processes and from combustion, such as municipal and medical waste incineration and backyard burning of trash. When consuming edible POPs-contaminated foods, the POPs accumulate in fatty tissue and can be passed on to children through breastfeeding [28].

When eating POPs-contaminated foods, the POPs accumulate in fatty tissue. Mothers pass POPs from their own bodies to their offspring. POPs contamination can lead to cancers and tumors, neurological disorders, immune suppression, reproductive disorders, and other diseases, including increased incidence of type 2 diabetes, endometriosis, hepatitis, and cirrhosis, relating to the chemical’s persistence, bioaccumulation, potential for long-range environmental transport, and adverse effects on human health or the environment.

International-auspice committees can evaluate whether the substance is likely, because of its long-range environmental transport, to lead to significant adverse human health and/or environmental effects and therefore if it warrants global action while developing a risk management evaluation that reflects the socioeconomic considerations associated with possible control measures [28]. Based on this, any party may nominate a chemical for evaluation. Each stage (criteria, risk profile, risk-management evaluation) typically takes one year, but some chemicals progress more slowly if the Persistent Organic Pollutants Review Committee (POPRC) requires additional time to gather and review relevant information. Since 2005, the COP has added 19 POPs to the original dirty dozen that are now controlled under relevant conventions [28].

Among the most frequently observed obstacles is that by creating the POPRC, the Stockholm Convention attempted to separate the scientific and technical consideration of the nominated POPs—the purview of the POPRC—from the political concerns of parties, which are discussed by the COP. In essence, the POPRC addresses whether the convention can control a substance and how it would do so. Then, the COP decides if it should. In this context, new challenges emerged, as the convention shifted to the evaluation of toxic chemicals still in widespread production and the use of “live” chemicals, especially with regard to socioeconomic considerations in the risk-management evaluation phase. This transition from “dead” to “live” chemicals illustrated the challenges of recommending policy responses to protect human health and the environment from chemicals that are still being used in applications, such as fire-fighting foams, which are important for human safety [28].

Another critique was related to living chemicals since the POPRC is forced to consider both the scientific and the socioeconomic implications of its recommendations to eliminate or reduce chemicals, including recommendations to allow some uses of the chemical to continue for a limited time when the use of safe alternatives chemicals is not economically or technically feasible. At the political level, however, the COP has sometimes agreed to allow additional uses of these live chemicals for longer time periods than the POPRC recommended, which delays the elimination or reduction in POP production and use [28].

Typical challenges are related to continuing implementation tasks which have to be addressed. For example, many governments still need to strengthen the legal, administrative, and other domestic measures to control POPs, including the development or revision of national legislation and regulations on POPs and their waste [28]. Governments need to strengthen environmentally sound management of POP waste by taking appropriate measures to manage stockpiles and wastes, particularly obsolete pesticides, in an environmentally sound manner to ensure they do not enter recycling streams. Governments also need to strengthen awareness-raising and information exchange, including engaging with populations most at risk of exposure to POPs [28].

The establishment of compliance procedures and mechanisms and strengthening information collection, especially on national inventories of the production, use, and releases of POPs, is needed. In addition, a perennial problem for all multilateral environmental agreements is the need to provide technical assistance and financial resources to developing country parties and parties with economies in transition. This is particularly important under the Stockholm Convention because these parties need assistance for the management and elimination of the POPs listed under the Convention [28]. 

Regulations targeting POPs have succeeded in reducing levels of POPs in humans and the environment. For the initial POPs, concentrations measured in the air and in human populations have declined and continue to decline or remain at low levels due to restrictions on POPs, some of which predated the Stockholm Convention and are now incorporated in it. For the newly listed POPs, concentrations are beginning to show decreases, although, in a few instances, increasing and/or stable levels are observed. But the Convention cannot afford to rest on its laurels, as these “forever chemicals” will take years to eliminate. And while they are still in the environment, human health and ecosystems remain at risk [28].

While pesticides have become a global solution for managing pests and diseases in agriculture, their excessive use in agriculture has detrimental effects on our environment, endangering the safety of our food supply and standing as a paramount concern in today’s world [29]. Therefore, while to the advantageous operation of pesticides, they also have the ability to kill plants and damage soil, potentially leading to a fall in both plant numbers and soil quality. These operational constraints of the in-field application of pesticides have been partially overcome by employing sophisticated mathematical modeling techniques, enabling researchers to obtain a comprehensive examination of their adverse effects [29]. In particular, the use of delay differential equations can capture the long-term effects of pesticides on plants and soil health. These multi-parametric models can also alter pesticide dosages and application rates on plants and soil, exposing crucial elements of determining pesticide uses and forecasting their environmental implications, offering a visual understanding of the complex interaction between pesticides and ecological processes [29].

In terms of agricultural socialization services, contemporary economies are grappling with the overuse of pesticides, and, among them, China emerges as a key consumer and producer, facing substantial challenges in environmental and agricultural sustainability. Therefore, there is an imperative need to determine how agricultural services can foster eco-friendly pesticide use among farmers [30]. It was shown that agricultural business services significantly boost green pesticide adoption, outperforming agricultural technology and input sectors. Such agricultural-oriented socialization services can increase the use of safer pesticides and the recycling of their containers by 11.6% and 6%, respectively [30]. However, it cannot undermine the pivotal role of farmers’ satisfaction and the inseparable regular internet use of today’s lifestyle in order to enhance the contemporary trend for low-toxicity pesticides and recycling programs, creating tailored policies for diverse agricultural needs and implementing resource management and incentive mechanisms towards promoting practices and strategies in favor of sustainable agriculture by balancing environmental health with economic viability [30].

The sources of the internet are not always directed to find good and sustainable agricultural practices of pesticide uses but also to be familiarized with the adverse health effects, such as those of pesticide allergy-related illnesses or pesticide exposure incidents, which underscore the need for safer and more effective pest management strategies [31]. Among the sensitive and vulnerable social groups of pesticide exposure are childcare administrators who ultimately protect children’s health by protecting children from pests and pesticide exposures in childcare centers. Here, the research has focused on evaluating and reducing the use of pesticides in K–12 schools, and for this, the role of web-based training can effectively supplement in-person childcare conferences and workshops by collecting a snapshot of pesticide use in childcare facilities [31].

The broader role of didactic, pedagogic education for a range of pesticide applications and the potential environmental impact of pesticides was the research objective of an interesting topic for science education (that of the chemistry course) [32]. It is also impactful when conventional pesticide use is contrasted with current chemistry research efforts to develop alternatives based on the ideas of green chemistry. At a secondary education level, a debate on the use of pesticides, especially among the public media, when it comes to topics such as organic farming, bee mortality, and the use of glyphosate has been literature-raised [32]. The authors of this study investigated the potential relevance of pesticides for chemistry education in connection with education for sustainable development. For this, a brief outlook on pesticides in science teaching and connecting the topic to socio-scientific issue-based chemistry education was further investigated in the form of a case study that developed a lesson plan for secondary school students. The definition of pesticides was followed by the development of green pesticides as potential alternatives to current products. Subsequently, video vignettes of a scientist introduced the topic to students. Finally, both glyphosate as a conventional, industrial pesticide and orange oil as an example of a green pesticide were compared using spider chart diagrams. Such a modeled course did a cyclical design from a group of ten chemistry teachers using participatory action research, aiming to support secondary school chemistry student teachers, and then was tested in five German secondary school classes and was especially helpful to the learners, most of whom felt that they were able to understand the controversy surrounding pesticides [32].

### 4.3. Overview of Applications of Green Pesticides

From an experimental perspective of analysis, the study of Viscusi et al. [33] is presented, in which the hemp fibers (HFs) were used as filler in pectin to produce biocomposites. The study aimed to improve the compatibility of the filler with the polymeric matrix, while the HFs were treated through a mechano-chemically assisted alkaline attack [33]. The effect of the treatment time on the morphology of the HFs was investigated through atomic force microscopy and infrared spectroscopy. For this, the green pesticide of cinnamic acid was added to the composites in order to test the capability of such materials to act as potential devices in the agricultural field. Aside from the analysis of the thermal, mechanical, barrier properties, and surface energy, the findings showed that the release of cinnamic acid was associated with the fibers’ treatment time, making the design and fabrication of a pot prototype of a plausible green functional container for plants to be implanted in the ground important [33].

Another experimental-based analysis referred to the resurgence of interest in natural substances and their progressive affirmation in the market opening doors for novel marketed products with intrinsic original approaches. Evident examples are in the food supplement sector as well as in a mix of natural substances applied. Towards the protagonist role of technology and the capacity to throw novel opportunities out of the normal landscape, most future technological developments rely on nanotechnology [34]. In this technological context, the green synthesis of nanopesticides is considered cheap and environmentally friendly since there is no need to employ highly toxic chemicals or elevating energy inputs. Therefore, the role of nano-based pest control can be even more important in near-future research [34].

Among other factors, a representative parameter of investigation in laboratory-scaled research is the pH profile. For completeness reasons, there are presented two cases, one referring to green pesticides and another referring to conventional chemical pesticides. In the green pesticide-referred study the gelatin beads reinforced with natural hemp hurd were produced as pH-sensitive devices for the release of eugenol as a green pesticide [35]. The composite beads, having a 1 mm mean diameter, were obtained by polymer droplet gelation in sunflower oil. The observed thermal properties showed no noticeable difference after the introduction of hemp hurd [35]. In addition, the swelling and dissolution phenomena of gelatin beads were studied as a function of pH. A rising of swelling of gelatin beads was observed as the pH increased up to 2.3 g/g, 9.1 g/g, and 27.33 g/g at a pH of 3, 7, and 12, respectively. Moreover, the dissolution rate changed from 0.034–0.077 h^−1^ at the range of pH 3–pH 12. In this study, the release kinetics of eugenol were determined at different pH conditions. The eugenol release behavior demonstrated high sensitivity to the pH release medium, thus allowing for tuning such devices as green pesticide release systems in soils with different levels of acidity/basicity [35].

From the conventional chemical pesticides viewpoint, a study is presented in which the effect of ionic strength on the adsorption of alachlor, trifluralin, and prometryn on Amberlite XAD-4 polymeric resin was studied [25]. In this study, static adsorption experiments were carried out at pHs of 3–6.5 and ionic strengths of 0.01–2 M at 20 °C. It was observed that by increasing the ionic strength, the adsorption of herbicides can be significantly increased. In addition, the reported alteration of adsorption mechanisms was attributed to ionic strength, especially due to the reduction in repulsive forces at the resin-herbicide interface and between adsorbing herbicide molecules since both resin and herbicides have a polar ring as part of their structure [25].

Moreover, a literature-reported strategy for green pesticides was based on the enhanced pesticidal activity and biocompatibility of the chitosan nanocomposite prepared with biocompatible polymer chitosan—insecticidal metabolites derived from the potential fungal biopesticidal agent Nomuraea rileyi [36]. The chitosan nanocomposite was prepared with metabolites and thus acquired by the ionic gelation method. It was noted that the synthesized nanocomposite sustained high stability and uniformly dispersed particles with high loading and entrapment efficiency [36]. At this point, it is noteworthy that chitosan nanocomposite treatment did not induce any toxic effect on the developmental stages of zebrafish [36]. Moreover, hemolysis was also not recorded in the nanocomposite treatment, implying that insecticidal metabolite-fabricated chitosan nanocomposites are promising candidates for pest control against economically important insect pests without affecting non-target organisms [36].

Among the well-known invertebrate fungal pathogens that play a significant role in controlling many agricultural pests and human disease vectors is that of Metarhizium anisopliae (Hypocreales: Clavicipitaceae, M. anisopliae) [37]. M. anisopliae is typically used as a chemical in dry or liquid formulations of large numbers of aerial conidia. The conidia can directly infect arthropod pests by penetrating their cuticular layer [37]. Therefore, the availability of the complete genome of M. anisopliae and capable techniques for its transformation have emerged potential applications and uses as a pest controller, thus necessitating more in-depth research, firstly, to improve fermentation and formulation technologies and, secondly, to promote the widespread acceptance and usefulness of M. anisopliae as a cost-effective fungal biopesticide [37]. Among the critical factors of investigation are that of molecular approaches to increase virulence and efficacy. Future studies of this fungus pathogen can be driven to demonstrate that M. anisopliae is proven as an efficient biocontrol agent, offering prosperous future research and development [37].

Over-reliance on synthetic pesticides in insect pest control has caused widespread public and scientific concerns for human health and the environment, especially since many insect pests have already developed resistance to conventional pesticides and their products [38]. For this, there is considerable interest in the development of alternative control methods for insect pest management. Green pesticides have a great potential to be used as an alternative tool to synthetic pesticides for insect pest management in crop production, thereby reducing threats to natural ecosystems and human health caused by the over-application of conventional synthetic pesticides [38]. From a geographical perspective of analysis, it is noteworthy to focus on China, where researchers recognized that the use of green pesticides is a key determinant in reducing the use of conventional pesticides and promoting environmental and food safety; however, the effectiveness of green pesticide use policies at the national level falls short of expectations. The existing research mainly examines the policy promotion issues of green pesticide use from a static and single-agent perspective. However, green pesticide use behavior is a dynamic process influenced by multiple factors, including the government, farmers, and consumers [39]. Proposed methodologies should pay attention to the difference in the individual endowment of farmers, improve the pertinence of technology promotion, provide policy-oriented green pesticide insurance products, and increase the technical support from the government [40]. Specific policies undertaken by the Chinese government should include substantial strengthening of ecological subsidies and market supervision, guidance of consumers’ green consumption behavior, and encouragement of farmers to use green pesticides [39].

From an economics–managerial perspective of analysis, the role of such novel technological approaches is also a cost–benefit issue since the estimation of about 2.5 million tons of pesticides are used on crops each year, and the worldwide damage caused by pesticides reaches USD 100 billion annually. In response to this contentious global and multifaceted problem, natural products are introduced as an excellent alternative choice to synthetic pesticides, reducing negative impacts on human health and the environment. These natural problems are also moving future research towards green chemistry processes, coupling with the development of new crop protection tools with novel modes of action. All of them make the discovery and commercialization of natural products as green pesticides sound like ideal alternatives to chemical pesticides [41]. Among the advantageous characteristics of green pesticides are their attributes as eco-friendly, economic, target-specific, and biodegradable. In this context, various plant essential oils showed a broad spectrum of activity against pest insects and plant pathogenic fungi ranging from insecticidal, antifeedant, repellent, oviposition deterrent, growth regulatory, and antivector activities [41].

From a commercial-trading perspective of analysis, among the most frequently reported studies on green pesticides are those of essential oils (EOs) from the flavor and fragrance industries, which are promising indicators to fast-track commercialization of essential oil-based pesticides [41]. Though well-received by consumers for use against home and garden pests, these green pesticides can effectively operate in agricultural fields for organic food production, especially concerning the retarded development of many synthetic pesticides of essential oil-based pesticides due to the complex mixtures of constituents that characterize pesticides based on plant essential oils or their constituents that have demonstrated efficacy against a range of tests to obtain the wanted properties as pests in a stored product [41]. Subsequently, pesticides based on plant essential oils are promising solutions to a variety of ways to control a large number of pests. Among the most well-known essential oil constituents are limonene, pulegone, citronellal, and 1,8-cineole, all being active ingredients of commercially available flea shampoos, mosquito repellents, and different agrochemicals [41].

EOs are the volatile constituents of chemical compounds with multiple modes of action that enhance their activity due to the synergistic effects of the constituents. Most EOs are serving as chemical messengers for post-harvest disease management [42]. The main property of EOs is their volatility, making them suitable fumigants and for the protection of post-harvest diseases of horticultural crops in protected environments. In addition, EOs sustained proven utility for the management of pests and diseases in agricultural fields and their crop yields. The existence of active constituents, monoterpenes, and sesquiterpenes has shown contact toxicity to post-harvest insect pests, but their utility is widely due to their sublethal behavioral effects as deterrents and repellents [42,43]. Moreover, EOs and their active constituents sustain potential bio-efficacy against insect pests when tested in the laboratory, but not all of them are broadly suitable in flavor and fragrance industries for developing pesticidal formulations. Such suitable EOs belong to the family Asteraceae, Myrtaceae, Lamiaceae, Lauraceae, Poaceae and have already developed a market presence in some countries as essential oil-based pesticides [42].

Similar functionality of EOs was demonstrated by Chaudhari et al. [44] according to whom many essential oils (EOs) and their bioactive compounds have received particular attention for application as botanical pesticides since they exhibited high insecticidal efficacy, a diverse mode of action, and favorable safety profiles on the mammalian system as well as to the non-target organisms [44]. Moreover, today’s situation of controlling storage insect pests is largely based on synthetic pesticides. However, due to fast-growing resistance in the targeted insects and the negative impact on humans and non-target organisms as well as the environment, there is an urgent need to search some safer alternatives to these xenobiotics [44]. These authors stressed that in spite of promising pesticidal efficacy against storage pests, the practical application of EOs and their bioactive compounds in real food systems remain rather limited because of their high volatility, poor water solubility, and susceptibility towards degradation [44]. Other characteristics of the green pesticides in alignment with their EO capabilities are selectively presented below:

- EOs do not show toxicity against mammals and fishes due to the absence of target sites in these organisms. A few essential oils are exempted from pesticide registration, being used as seasoning agents in food and beverage products. In parallel, these EOs can be perceived as potential green pesticides against agricultural pests and support organic food production [43].
- Medicinal plants have been playing a significant role in the management of several plant diseases. Plentiful medicinal plants contain several components and pigments that show toxicity against various microbes, including fungi, which are responsible for spreading plant diseases [45]. Phytochemicals isolated from medicinal plants are key ingredients in green pesticides. The frequent use of chemical fungicides results in various environmental problems, all related to the pursuit of sustainability. Therefore, research focuses on exploiting the extracts of medicinal and aromatic plant species. Plant protection involves the secondary metabolites secreted by medicinal plants used throughout the world, which are accountable for their biological characteristics [45].
- EOs’ impact on wheat flour quality, the volatile compounds retained by the treated substrate, and acetylcholinesterase (AChE) activity showing a long time, up to 60 days, of storage enabled the retention of significant amounts of menthone. Thereafter, EO substrate treatment changed flour moisture and protein content, and the EO exhibited insecticidal activity via inhibiting acetylcholinesterase activity [46].
- Among the EOs studied, in order to find effective, biorational, and eco-friendly pest control tools, the EO of Cupressus sempervirens var. horizontalis was produced using hydrodistillation, while typical analytical techniques contained gas chromatography, specifically, using flame ionization detection [47].

## 5. Conclusions Implications and Future Research Works

Based on the collected research studies, it is obvious that the data demonstrated an excessive use application of pesticides in the agricultural sector but also a reported and proven causality of human fertilization implications with this abundant presence of pesticides in natural sources and edible foods. In addition, since a great variety of analytical techniques have been available and interconnected with different processes of experimental or pilot scales, there is promising literature production that the most frequently and mainly applied methods in conventional laboratory experiments can be further developed, thus enhancing our understanding of the mechanisms of pesticide fate when applied in agricultural fields and when edible crops are the transformers from land to dish.

Therefore, non-conventional innovative technologies can be developed in order to support the scalability of modeling-oriented applications towards large-scale and in-field innovative solutions for drinking water treatment and pesticide removal (especially from aqueous solutions). Among such innovative technologies are those related to synthetic or natural polymers, as well as to the carbonaceous materials of high adsorptive capacities of pollutants in aqueous solutions. Technological advancements in polymer science can advance the production of highly porous polymers that have similar adsorption capabilities to that of activated carbon [13,25,26,48]. Indicative future research works can be concentrated on the following issues:

- The reduction potential of fertilizer and pesticide input without compromising high agricultural yields. In such a case, rice grain applies a double rice cropping system in the subtropical hilly regions, like that of China, where rice is among the most important nutrition crops for population nutrition [17].
- It is a fact that at present, the phenomenon of excessive pesticide residues in vegetables is prominent, causing widespread concern among all sectors of society. It is important to excavate the influencing factors in the farmers themselves, the government, market, and society that affect vegetable farmers’ green pesticide application behavior and to clarify the influence mechanism of influencing factors on vegetable farmers’ green pesticide application behavior. In this context, future research studies have to reveal various factors in vegetable farmers’ green pesticide application behavior (GB). For this, government supervision and regulation (GR) and market adjustment guidance (MG) are considered external factors that regulate the strength of the relationship between GM and GB. Therefore, it is necessary to further strengthen the reference and the normative role of society in vegetable farmers’ GB in order to draw driving strategies for vegetable farmers’ GB and to simultaneously ensure both improvements in the quality of pesticide application and vegetable safety [49,50].

## Figures and Tables

**Table 1:** Profile of key aspects and information contained per document retrieved. Source: authors’ own study based on [3,8,9,12,13,14,15,16,17,18,19,20,21].

Ref. Item #	Pesticide/Pollutant	Aim–Experimental–Results
[12] Kholkute et al., 1993	aldrin; atrazine; endosulfan; endrin; heptachlor; herbicide; hexazinone; metribuzin, propachlor; simazine	Two herbicide mixtures, M-HERB-1 and M-HERB-2, applied in vitro fertilization (IVF) in mouse trial. Oocytes contained in cumulus masses collected from superovulated B6D2F1 mice were cultured in media containing either the pesticide mixture M-HERB-1 or M-HERB-2, to which capacitated sperm were added. Oocytes were assessed for fertilization 20–24 h after insemination. The pesticide mixture significantly affected the IVF rate and caused an increased incidence of abnormal embryos and degenerative oocytes at the 10 μg concentration. Both herbicide mixtures failed to show any significant effect on fertilization but did show a trend towards an increased incidence of degenerative oocytes following culture.
[3] Tielemans et al., 1999	Not specifying the applied pesticides. It is a survey-based study. The survey was based on a questionnaire among respondents working in the fields of livestock farming, gardening.	A total of 836 couples who sought in vitro fertilization (IVF) treatment between 1991–1998. Questionnaire information was collected for exposure of the spouses to organic solvents, metal dust or fumes, welding fumes, and pesticides. Paternal pesticide exposure decreased the sperm fertilizing ability in vitro. However, the exposition of multiple pesticides with various active ingredients made it impossible to identify the hazardous chemical action.
[13] Saitoh et al., 2001	Non-specific pesticides were applied. Experimental session of rice was grown by pesticide-free organic cultivation with only about 10% yield reduction.	The effects of organic cow and chicken manure fertilization and pesticide application on the yield of rice (cultivar Nipponbare) were examined in 1990–1999 in the paddy field of Okayama University. In this study, the relative reduction in organic pesticides by 10% was observed compared to chemical fertilizers. This reduction can be attributed to competition with weeds, especially monochorea, for nutrients. Yearly fertilization of manure increased the total carbon and nitrogen concentration of the soil, supporting soil fertility.
[14] Wang et al., 2006	Endosulfan, dieldrin, dndrin, HCB, HCH, DDE, DDT, DDD	Soil samples with three fertilization treatments: no fertilizer, corn straw, and farm manure were collected from Lou soil (Eumorthic Anthrosol classified using Chinese Soil Taxonomy) in northwestern China. Organochlorine pesticides (OCPs) were detected in all soil samples except δ-HCH, and their total concentrations ranged from 159.31 ± 9.00 to 179.77 ± 2.58 ng/g with an order of HCHs > DDTs > (dieldrin + endrin) > HCB > α-endosulfan. Among all the compounds, γ-HCH sustained the highest concentration followed by p, p′-DDE. It was also reported lower DDT residues than samples without fertilizer at farm manure treatments, while corn straw and farm manure increased soil organic matter content and decreased the soil pH, enabling the retarding degradation of DDT in the soil.
[9] Lima et al., 2012	Carbamates, organochlorines, organophosphates, herbicides (Organochlorine and organophosphate pesticides of the aforementioned characteristic chemical groups)	The vegetable materials analyzed were the following: - Beet, including peel, stalks, and leaves of jack fruit, sweet potato, green papaya, lemongrass. - Three types of polyamines (PA): putrescine (Put), spermidine (Spd), and spermine (Spm), as well as nitrate and phenols content detection. - Nitrate content determination. - Total phenol content determination. - Higher concentrations of hydrolyzable polyphenols in organically grown vegetables. - Absence of pesticides glyphosate and atrazine in conventionally grown vegetables. - Higher nitrate content was observed in foods from conventional cultivation, except for the pulp of green papaya. Organic potatoes presented lower nitrite and nitrate content (about 80%) compared with conventional potatoes. Nitrate is observed due to conventional cultivation compared to organically derived plants. The only analyzed foods that contained pesticide residues (mainly organochlorine insecticides) were from crops that all originated from conventional fertilization productions.
[8] Muñoz-Leoz et al., 2012	Difenoconazole, deltamethrin, ethofumesate	The application of pesticides and fertilizers to soil was counted for 5 mg active ingredient kg^−1^ DW soil and 185 mg N kg^−1^ DW soil, respectively. After a time of 0, 7, 30, 60, and 90 days of incubation, soil samples were received in order to determine pesticide degradation rates and microbial properties. Experimental sessions revealed the interactions between the pesticides of difenoconazole: fungicide, deltamethrin: insecticide, ethofumesate: herbicide, as well as the fertilizers of NPK synthetic fertilizer, compost, all referring to the potential non-target effects of pesticides on soil microbial communities. After incubation phase, the pesticides of difenoconazole, deltamethrin, and ethofumesate in non-fertilized soils were degraded by 52, 85, and 93%, showing half-lives of 86, 36, and 29 days, respectively. In addition, there was a short-term inhibitory effect on microbial activity due to non-fertilized soils difenoconazole and deltamethrin but not in fertilized soils.
[15] Giraldo-Charria et al., 2019	Insecticides: Bifenthrin and Imidacloprid Fungicide: Carbendazim	Systemic insecticides and/or fungicides were applied through injections in the trunk of 15 tree species affected by the progressive deterioration of the crown in the urban forests of the Metropolitan Area of the Aburrá Valley. The experimental treatments followed two sessions of pesticide applications 1 and 2. Environmental stress and climatic variability were associated with the critical parameters of planting site, traffic flow of the site, the wood density of the species, and time. The applied treatments did not affect the recovery of the species, while insects and pathogens showed an opportunistic hazard factor once trees were affected.
[16] Gurushankara et al., 2012	Insecticides: Malathion and Methyl parathion	Considering the possibility of exposure of amphibian eggs to pesticides in agro-ecosystems, authors determined the effect of different concentrations of the malathion and methyl parathion insecticides on the eggs of Fejervarya limnocharis (it is an Indian Cricket frog species). Time of exposure, amount of sialic acid, and egg hatchability were determined. Insecticides can alter sialic acid content in jelly coat of amphibian eggs, leading to inhibition of sperm penetration.
[17] Liu et al., 2020	Herbicide: Butachlor Molting hormone: Tebufenozide Insecticide: Emamectin	Application of chemical fertilizer and pesticides in investigating in-field N and P runoff losses paddy water. N and P runoff losses were performed in the double rice cropping system under the interaction of chemical fertilizer and pesticide, China. Early rice season showed higher N and P runoff losses than late rice season due to the fact that N and P fertilizer use efficiencies were lower; thus, paddy water N and P concentrations were higher in the early rice season. N and P runoff losses were influenced by chemical fertilization but not by pesticide application.
[18] Naas and Saleh, 2020	Nitrates-contained chemical pesticides (not specified or defined)	The concentration of nitrates using Green Test Eco device for fruits and vegetables of various origins (greenhouses and open agriculture) in Iraq measured the percentage of toxic residual substances after applying fertilizers and chemical pesticides. The Green Test Eco is a mobile device that performs rapid measurements of nitrite ratios in different fruits having an allowable/permissible standard limit level for six fruit crops. Nitrate average measured value for imported fruits, local fruits grown inside greenhouses, and local vegetables cultivated in the open field. Nitrates average was less in the fruits planted in the greenhouse, while the local fruits planted in open fields were edible, sustaining less than the allowable standard level of nitrite.
[19] Kumari et al., 2021	Organophosphate pesticide: Quinalphos (QP)	Adult Swiss albino male mice were orally administered 0.25, 0.5, and 1.0 mg/kg of QP (Ekalux 25 E.C.) for ten consecutive days, and the reproductive function was assessed at 35 and 70 days after QP treatment. In this study, the reported quantities were that of high oestradiol, low testosterone, aromatase, and cytochrome P450 transcript levels increase. Significant decreases in sperm functional competence and fertilizing ability, lower blastocyst rate, and poor blastocyst quality were reported when spermatozoa collected from QP-exposed mice were subjected to in vitro fertilization. These symptoms appeared to be mediated through elevated oxidative stress and altered steroidogenesis in testes. A QP slow clearance from tissue was detected even after 24 hr of administration.
[20] Wang et al., 2021	Benzimidazole fungicide: Carbendazim Organophosphate insecticide: chlorpyrifos Neonicotinoid insecticide for chemical pesticide control: Thiamethoxam	Different fertilization and pesticide application methods were used in sweet potato production process to analyze the advantages of layered fertilization and pesticide application methods in sweet potato ridge cores. Fertilization and pesticide application of sweet potatoes is a key process in sweet potato planting. Machinery’s lack of fertilization and pesticide application of sweet potatoes impeded to mechanization of the whole process but also restricted the development of the sweet potato industry. Jointly applying sweet potato fertilization and pesticide application suffered from over-fertilization, inability to deploy fertilizer in the core and layers, and few specific equipment for fertilizer application.
[21] Zhang et al., 2023	Not specifying the applied pesticides. It is a survey-based study.	The research objective of this study is the outsourcing of fertilization and pesticide application for the growth of wheat production. Farmers’ behavioral decisions regarding outsourcing pesticide application (OPA) were determined by social interactions. In this study, it was noted that if a farmer’s neighbor chose to outsource, this action further affected farmer’s outsourcing behavior. In addition, the decision for farmer outsourcing in a village reflected the local availability of outsourcing, the cost of outsourcing, as well as the market development of outsourcing to some extent. In addition, selecting reasonable instrumental variables to indirectly affect the technical efficiency (TE) of the respondents’ wheat production through other pathways should be considered jointly with training for farmers to enhance their outsourcing knowledge in choosing the appropriate outsourcing times to improve TE.

**Table 2:** Stereo-chemical structure, IUPAC names, categories, pKa or log Kow values of the 35 reported pesticides. Sources: synthesized by authors based on [8,9,12,14,15,16,17,19,20].

Pesticide Name	Molecular Type/IUPAC Name	Category/Description of Functionality	Acid Dissociation (pKa) or Octanol/Water Partitioning (log Kow)	Ref Item # in Table 1
Aldicarb	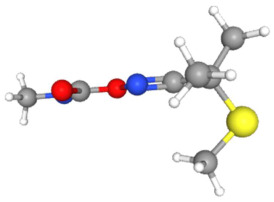 [(E)-(2-methyl-2-methylsulfanylpropylidene)amino] N-methylcarbamate C_7_H_14_N_2_O_2_S	Aldicarb (banned) Insecticide Aldicarb is an oxime carbamate.	(log Kow) 1.13	[9]
Aldrin	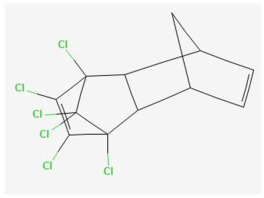 (1S,2S,3S,6R,7R,8R)-1,8,9,10,11,11-hexachlorotetracyclo[6.2.1.13,6.02,7]dodeca-4,9-diene C_12_H_8_Cl_6_	Insecticide (banned) Aldrin, liquid appears as a solution in oil of aldrin, a noncombustible water-insoluble solid. Mixed with a flammable carrier solvent Aldrin was banned for all uses (except to control termites) by EPA in 1974, being responsible for environmental damage and human implications. All uses banned by EPA were regulated in 1987.	(log Kow) 4.50	[12]
Atrazine	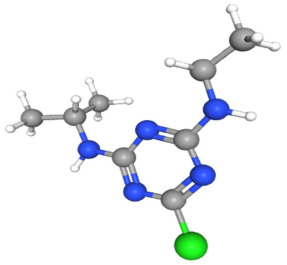 6-chloro-4-N-ethyl-2-N-propan-2-yl-1,3,5-triazine-2,4-diamine C_8_H_14_ClN_5_	Atrazine (banned) Herbicide Atrazine is a diamino-1,3,5-triazine that is 1,3,5-triazine-2,4-diamine substituted with a chloro group at position 6 while one of hydrogens of each amino group is replaced by an ethyl and a propan-2-yl group, respectively. EPA restricts the applicability of atrazine only to trained people who are allowed to spray it, making it suitable on farms but also on highway and railroad rights-of-way.	(pKa) 1.70	[9,12]
Bifenthrin	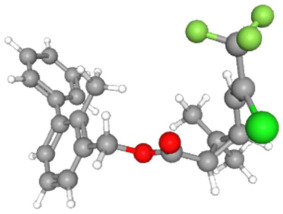 (2-methyl-3-phenylphenyl)methyl (1R,3R)-3-[(Z)-2-chloro-3,3,3-trifluoroprop-1-enyl]-2,2-dimethylcyclopropane-1-carboxylate C_23_H_22_ClF_3_O_2_	Bifenthrin (banned) Pyrethroid insecticide Bifenthrin is a carboxylic ester obtained by formal condensation of cis-3-(2-chloro-3,3,3-trifluoroprop-1-enyl)-2,2-dimethylcyclopropanecarboxylic acid and [(2-methyl-1,1′-biphenyl)-3-yl]methanol. It is an organochlorine compound. Its functionality is related to a cis-chrysanthemic acid.	(log Kow) 6.0	[15]
Butachlor	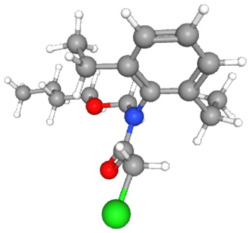 N-(butoxymethyl)-2-chloro-N-(2,6-diethylphenyl)acetamide C_17_H_26_ClNO_2_	Acetanilide herbicide Butachlor is an aromatic amide, an organochlorine compound, and a tertiary carboxamide. Butachlor is a selective herbicide used globally in the cultivation of corn, soybean, and other crop cultures.	(log Kow) 4.5	[17]
Carbendazim	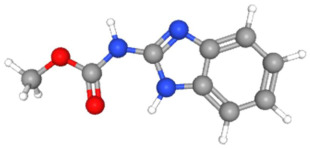 methylN-(1H-benzimidazol-2-yl)carbamate C_9_H_9_N_3_O_2_	Carbendazim (banned) Benzimidazole fungicide Carbendazim is a member of the class of benzimidazoles being applied to crops, including bananas, cereals, cotton, fruits, grapes, tobacco, and vegetables, being an antifungal agrochemical.	(pKa) 4.48	[15,20]
Carbofuran	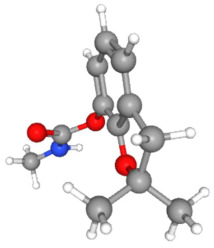 (2,2-dimethyl-3H-1-benzofuran-7-yl) N-methylcarbamate C_12_H_15_NO_3_	Carbofuran (banned) Acaricide and insecticide Carbofuran is a carbamate ester and a member of 1-benzofurans, being acetylcholinesterase and cholinesterase inhibitor, as well as acaricide agrochemical and nematicide.	(log Kow) 2.32	[9]
Chlorpyrifos	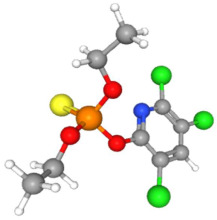 diethoxy-sulfanylidene-(3,5,6-trichloropyridin-2-yl)oxy-λ^5^-phosphane C_9_H_11_Cl_3_NO_3_PS	Organophosphate insecticide Chlorpyrifos is an insecticide that is a white crystal-like solid with a strong odor, being mainly applied in homes (control of cockroaches, fleas, termites) and on farms (control ticks on cattle being sprayed to control crop pesticides).	(log Kow) 4.96	[20]
Deltamethrin	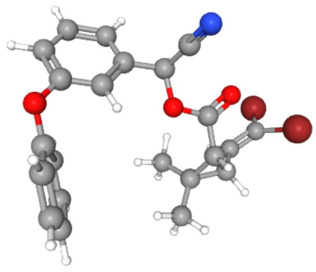 [(S)-cyano-(3-phenoxyphenyl)methyl] (1R,3R)-3-(2,2-dibromoethenyl)-2,2-dimethylcyclopropane-1-carboxylate C_22_H_19_Br_2_NO_3_	Insecticide Deltamethrin is a cyclopropanecarboxylate ester obtained by formal condensation between 3-(2,2-dibromovinyl)-2,2-dimethylcyclopropanecarboxylic acid and cyano(3-phenoxyphenyl)methanol. Deltamethrin holds the active insecticide functioning of the pro-insecticide tralomethrin.	(log Kow) 6.20	[8]
DDD (p,p’)	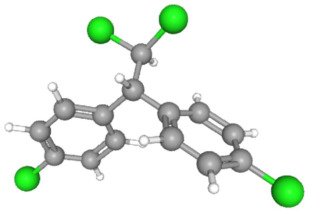 1-chloro-4-[2,2-dichloro-1-(4-chlorophenyl)ethyl]benzene C_14_H_10_Cl_4_	DDD (p,p’) (banned) Organochlorine insecticide DDD is a chlorophenylethane that is 2,2-bis(p-chlorophenyl)ethane substituted with two chloro groups at position 1. It is a metabolite of the organochlorine insecticide, DDT. It has a role as a xenobiotic metabolite. DDD is applied to medically treat cancer of the adrenal gland.	(log Kow) 5.061–6.217	[14]
DDE (p,p’)	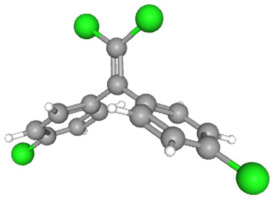 1-chloro-4-[2,2-dichloro-1-(4-chlorophenyl)ethenyl]benzene C_14_H_8_Cl_4_	DDE (p,p’) (banned) When ethylene is substituted by two 4-chlorophenyl groups at position 1 and two chlorine atoms at position 2 at a chlorophenylethylene, this forms the DDE. It is a persistent organic pollutant and a human xenobiotic metabolite. DDE is a breakdown product of DDT and has not been commercially marketed.	(log Kow) 5.69–6.96	[14]
DDT (p,p’)	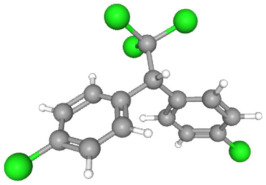 1-chloro-4-[2,2,2-trichloro-1-(4-chlorophenyl)ethyl]benzene C_14_H_9_Cl_5_	DDT (p,p’) (banned) Organochlorine insecticide DDT is a chlorophenylethane that is 1,1,1-trichloro-2,2-diphenylethane substituted by additional chloro substitutes at position 4 of the phenyl substitutes.	(log Kow) 6.91	[14]
Difenoconazole	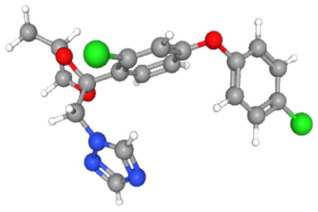 1-[[2-[2-chloro-4-(4-chlorophenoxy)phenyl]-4-methyl-1,3-dioxolan-2-yl]methyl]-1,2,4-triazole C_19_H_17_Cl_2_N_3_O_3_	Fungicide Difenoconazole is a member of the class of dioxolanes that is 1,3-dioxolane substituted at position 2 by 2-chloro-4-(4-chlorophenoxy)phenyl and 1,2,4-triazol-1-ylmethyl groups. Difenoconazole functions as a broad-spectrum fungicide of novel broad-range activity, which has been applied for seed treatment and foliar spray. It is a sterol 14alpha-demethylase inhibitor and an anti-fungal agrochemical.	(pKa) 1.1	[8]
Dieldrin	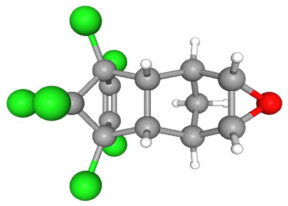 (1R,2S,3S,6R,7R,8S,9S,11R)-3,4,5,6,13,13-hexachloro-10-oxapentacyclo[6.3.1.13,6.02,7.09,11]tridec-4-ene C_12_H_8_Cl_6_O	Dieldrin (banned) Organochlorine insecticide Under the epoxidation of the double bond of aldrin, then, the organochlorine compound of dieldrin is produced. It is the active metabolite of the pro-insecticide aldrin. Specific functions of dieldrin are its xenobiotic and carcinogenic agency.	(log Kow) 3.692–6.2	[14]
Emamectin (B1b)	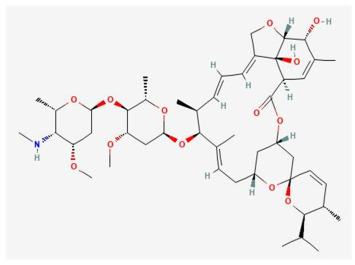 (1′R,2R,3S,4′S,6S,8′R,10′E,12′S,13′S,14′E,16′E,20′R,21′R,24′S)-21′,24′-dihydroxy-12′-[(2R,4S,5S,6S)-4-methoxy-5-[(2S,4S,5R,6S)-4-methoxy-6-methyl-5-(methylamino)oxan-2-yl]oxy-6-methyloxan-2-yl]oxy-3,11′,13′,22′-tetramethyl-2-propan-2-ylspiro[2,3-dihydropyran-6,6′-3,7,19-trioxatetracyclo[15.6.1.14,8.020,24]pentacosa-10,14,16,22-tetraene]-2′-one C_48_H_73_NO_13_	Insecticide Emamectin is an insecticide due to its chloride channel activation properties. It is derived from avermectin B1, also named “abamectin”, being a mixture of the natural avermectin B1a and B1b. Emamectin is produced by the bacterium streptomyces avermitilis, having toxicity for nematodes, arthropods, and several other pests. EPA approved Emamectin in prevention of emerald ash borer in ash trees. Emamectin is suitable for the eradication of fish lice and in fish farming.	(pKa) 4.2 and 7.6	[17]
Endosulfan	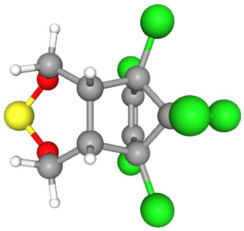 1,9,10,11,12,12-hexachloro-4,6-dioxa-5λ4-thiatricyclo[7.2.1.02,8]dodec-10-ene 5-oxide C_9_H_6_Cl_6_O_3_S	Endosulfan (banned) Cyclodiene organochlorine acaricide and insecticide, an agrochemical and a persistent organic pollutant Endosulfan is a cyclic sulfite ester that is 1,5,5a,6,9,9a-hexahydro-6,9-methano-2,4,3-benzodioxathiepine 3-oxide substituted with chloro groups at positions 6, 7, 8, 9, 10, and 10.	(log Kow) 2.23 3.55 3.62 3.13	[12,14]
Endrin	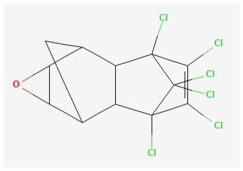 3,4,5,6,13,13-hexachloro-10-oxapentacyclo[6.3.1.13,6.02,7.09,11]tridec-4-ene C_12_H_8_Cl_6_O	Endrin (banned) Herbicide Endrin forms a white crystalline, odorless solid that has dissolved in a liquid carrier, being also water emulsifiable. It was marketable and used as a pesticide.	(log Kow) 3.209–5.339	[12,14]
Ethofumesate	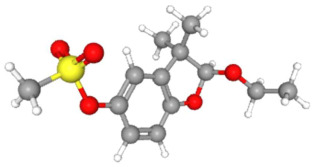 (2-ethoxy-3,3-dimethyl-2H-1-benzofuran-5-yl) methanesulfonate C_13_H_18_O_5_S	Pre- and post-emergence herbicide Ethofumesate is applied to grasses and as broad-leaved weed control in various crops.	(log Kow) 2.70	
Glyphosate	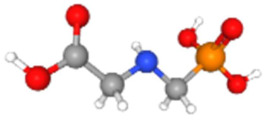 2-(phosphonomethylamino)acetic acid C_3_H_8_NO_5_P	Herbicide Glyphosate is the active ingredient in weed-killer products, such as that of RoundUp™. Glyphosate controls broad-leaf weeds and grasses and is applied as weed killer globally in farms and in home gardens and lawns.	(pKa) 5.60	[9]
HCB	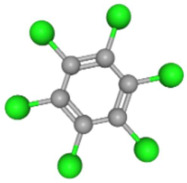 methylidyneborane C_6_Cl_6_	HCB (banned) Hexachlorobenzene fungicide HCB is characterized by its volatile and low aqueous solubility with a low potential for leaching into groundwater. Very persistent in soil systems. Low mammalian toxicity and high potential to bioaccumulate.	(log Kow) 3.93–6.42 5.175	[14]
HCH	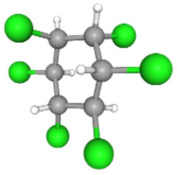 1,2,3,4,5,6-Hexachlorocyclohexane C_6_H_6_Cl_6_	HCH (banned) Insecticide HCH is a chemical compound that exists in eight manufacturing forms called isomers. The gamma-HCH (or γ-HCH) is named lindane and mainly functions as insecticide on fruit, vegetables, and forest crops.	(log Kow) 3.80	[14]
Heptachlor	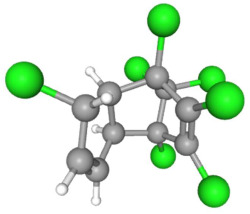 1,5,7,8,9,10,10-heptachlorotricyclo[5.2.1.02,6]deca-3,8-diene C_10_H_5_Cl_7_	Heptachlor (banned) Cyclodiene organochlorine insecticide functions as termite killer or controls ants and other insects in agricultural and domestic applications/contexts. It is also an agrochemical and antibacterial agent. Heptachlor is a cyclodiene organochlorine insecticide that is 3a,4,7,7a-tetrahydro-1H-4,7-methanoindene substituted with chlorine atoms at positions 1, 4, 5, 6, 7, 8, and 8.	(log Kow) 4.4–5.5 4.95	[12]
Hexazinone	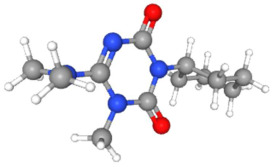 3-cyclohexyl-6-(dimethylamino)-1-methyl-1,3,5-triazine-2,4-dione C_12_H_20_N_4_O_2_	Herbicide Hexazinone is an organic compound that functions as broad-spectrum herbicide. Its main properties are its colorless solid texture, having some solubility in water, but a high solubility in most organic solvents, except alkanes. It is a member of triazines, and it is sold under the trade name Velpar^TM^.	(pKa) 2.2	[12]
Imidacloprid	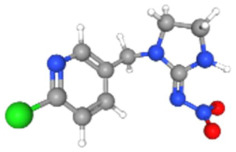 (NE)-N-[1-[(6-chloropyridin-3-yl)methyl]imidazolidin-2-ylidene]nitramide C_9_H_10_ClN_5_O_2_	Neonicotinoid insecticide Imidacloprid is a neonicotinoid, which is a class of neuro-active insecticides based on nicotine-building morphology. Sells under 10 different trade names as marketed pest control, seed treatment, and insecticide spray.	(log Kow) 1.20	[15]
Malathion	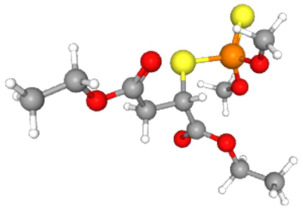 Diethyl dimethoxyphosphinothioylsulfanylbutanedioate C_10_H_19_O_6_PS_2_	Malathion (banned) Insecticide Malathion is a parasympathomimetic organophosphate compound, having the main function of insecticide for the treatment of head lice. Malathion action is targeted to the nervous system of the lice by irreversibly inhibiting the activity of cholinesterase and, subsequently, allowing acetylcholine to accumulate at cholinergic synapses to enhance cholinergic receptor stimulation and to cause the head lice’s death.	(log Kow) 2.36	[9,16]
Methyl parathion	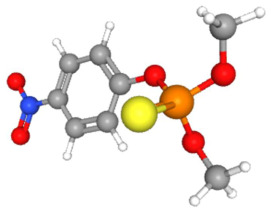 Dimethoxy-(4-nitrophenoxy)-sulfanylidene-lambda5-phosphane C_8_H_10_NO_5_PS	Insecticide Methyl parathion is a white crystalline solid, commonly dissolved in a liquid solvent carrier of pungent odor, slightly soluble to insoluble in water, but combustible liquid and highly toxic by inhalation, ingestion, and skin absorption.	(pKa) 7.15	[16]
Metribuzin	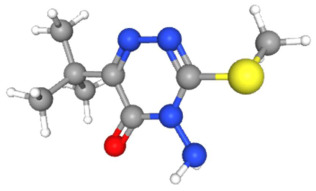 1,2,4–Triazinone C_8_H_14_N_4_OS	Metribuzin (banned) Herbicide that functions as a xenobiotic, an agrochemical, as well as an environmental contaminant. Metribuzin is a member of the class of 1,2,4-triazines.	(pKa) 1.00	[12]
Propachlor	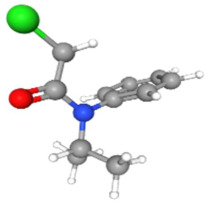 2-Chloro-N-isopropyl-N-phenylacetamide C_11_H_14_ClNO	Organochlorine herbicide Propachlor is an anilide that consists of 2-chloroacetanilide bearing an N-isopropyl substituent. It is an environmental contaminant and xenobiotic anilide.	(log Kow) 1.62–2.30 1.96	[12]
Putrescine	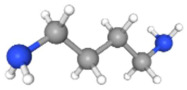 1,4-Diaminobutane C_4_H_12_N_2_	Fungicide Putrescine is a toxic diamine formed by putrefaction from the decarboxylation of arginine and ornithine.	(pKa) 10.8	[9]
Quinalphos	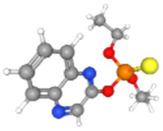 diethoxy-quinoxalin-2-yloxy-sulfanylidene-lambda5-phosphane C_12_H_15_N_2_O_3_PS	Organophosphate insecticide Organic thiophosphate insecticide. Acetylcholinesterase inhibitor, acaricide, and an agrochemical.	(log Kow) 4.4	[19]
Simazine	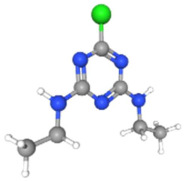 1,3,5-Triazine C_7_H_12_ClN_5_	Simazine (banned) Herbicide Simazine is a diamino-1,3,5-triazine that is N,N’-diethyl-1,3,5-triazine-2,4-diamine substituted with a chloro group at position 6. The “banned” prohibition of simazine (together with atrazine) is attributed to their endocrine disruptor effects and their ubiquitous water contamination.	(pKa) 1.62	[12]
Spermidine	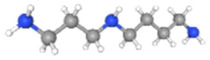 1,5,10-Triazadecane C_7_H_19_N_3_	Fungicide Spermidine is a triamine that is the 1,5,10-triaza derivative of decane. A fundamental metabolite, a geroprotector, and an autophagy inducer.	(pKa) 10.1, 9.1, 6.5 8.566	[9]
Spermine	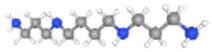 N,N’-bis(3-aminopropyl)butane-1,4-diamine C_10_H_26_N_4_	Fungicide Spermidine-derived biogenic polyamine found as a polycation at all pH values. Antioxidant, immunosuppressive agent, a fundamental metabolite.	-	[9]
Tebufenozide	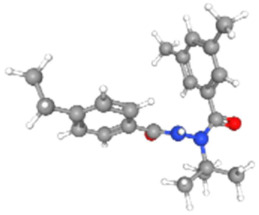 N-tert-Butyl-N’-(4-ethylbenzoyl)-3,5-dimethylbenzohydrazide C_22_H_28_N_2_O_2_	Insecticide functioning at molting hormone used widely against caterpillars Tebufenozide functions as a carbohydrazide, being a hydrazine having replaced its amino hydrogens by tert-butyl, 3,5-dimethylbenzoyl, and 4-ethylbenzoyl groups, respectively.	(log Kow) 3.90	[17]
Thiamethoxam	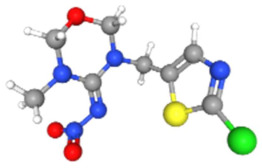 (NE)-N-[3-[(2-chloro-1,3-thiazol-5-yl)methyl]-5-methyl-1,3,5-oxadiazinan-4-ylidene]nitramide C_8_H_10_ClN_5_O_3_S	Thiamethoxam (banned) Neonicotinoid of broad-spectrum neonicotinoid insecticide. A nitro-oxazine and thiazole derivative.	(log Kow) 1.50	[20]

Notation: The colored chemical elements correspond to: C: grey; H: white; O: red; N: blue; Cl: green; P: orange; S: yellow.

## Data Availability

No data availability.
